# Signatures of a globally optimal searching strategy in the three-dimensional foraging flights of bumblebees

**DOI:** 10.1038/srep30401

**Published:** 2016-07-27

**Authors:** Mathieu Lihoreau, Thomas C. Ings, Lars Chittka, Andy M. Reynolds

**Affiliations:** 1 Department of Biological and Experimental Psychology, School of Biological and Chemical Sciences, Queen Mary University of London, Mile End Road, London E1 4NS, UK; 2 Rothamsted Research, Harpenden, Hertfordshire, AL5 2JQ, UK

## Abstract

Simulated annealing is a powerful stochastic search algorithm for locating a global maximum that is hidden among many poorer local maxima in a search space. It is frequently implemented in computers working on complex optimization problems but until now has not been directly observed in nature as a searching strategy adopted by foraging animals. We analysed high-speed video recordings of the three-dimensional searching flights of bumblebees (*Bombus terrestris*) made in the presence of large or small artificial flowers within a 0.5 m^3^ enclosed arena. Analyses of the three-dimensional flight patterns in both conditions reveal signatures of simulated annealing searches. After leaving a flower, bees tend to scan back-and forth past that flower before making prospecting flights (loops), whose length increases over time. The search pattern becomes gradually more expansive and culminates when another rewarding flower is found. Bees then scan back and forth in the vicinity of the newly discovered flower and the process repeats. This looping search pattern, in which flight step lengths are typically power-law distributed, provides a relatively simple yet highly efficient strategy for pollinators such as bees to find best quality resources in complex environments made of multiple ephemeral feeding sites with nutritionally variable rewards.

Constantly changing temporal and spatial distributions of resources provide complex challenges to foraging animals. Understanding how they explore space can give insight into how they find and selectively exploit these resources efficiently. Pollinators, such as bees, provide a good model for studying animal search behaviour, and as a consequence the impressive abilities of bumblebees and honeybees to exploit nectar and pollen resources for their colony have been investigated intensively^1^,^2^,^3^. The optimal foraging literature suggests that bees tend to use partially hard-wired movement rules that maximise energy gains, such as moving from bottom to top of vertical inflorescences^4^,^5^, making turning angles when visiting flowers within a patch contingent on recently experienced rewards^6^,^7^ or keeping constant arrival and departure directions when moving between different patches^8^. While this is an important first step, these studies typically assume that foragers are informed (at least partially) about the location and the profitability of available resources and thus do not to account for the actual process of searching and choosing which resources to exploit when an animal discovers an unfamiliar environment. Fast development of automated tracking technologies to record accurately animal movements across a variety of spatial and temporal scales is beginning to reveal that animal search strategies are often more complex than previously thought, depending on their sensory perceptual and cognitive abilities^9^,^10^.

The objective of this study was, for the first time, to map and characterize the flight patterns of bumblebee workers as they search for flowers arranged in a three-dimensional space. Previous observations of bees searching in two-dimensional arrays of flowers (where all food resources are at the same height relative to the ground) suggest that foragers adjust their flight speed and height based on the optical properties of flowers, such as colour contrast and size, to trade-off between rapid detection and reliable identification^11^. Here we examined whether these variations of flights patterns are characteristic of a more global search strategy for discovering new food sources, akin to that seen in the ‘orientation’ flights of bumblebees during which bees acquire spatial memories of the colony surroundings on their first foraging attempts outside the nest^12^. Novice bumblebee foragers typically fly in complex loops centred around the colony nest location, whose size tend to increase over time, so that a small number of loops make a substantial contribution of the total length of a search path. This increase in loop length is eventually curtailed by biological and environmental constraints, such as exhaustion of foragers or physical boundaries (e.g. treelines). This flight pattern resembles an optimal ‘Levy’ searching strategy, the hallmark of which is a loop-length distribution with a truncated power-law tail^13^. Unlike other finite-scale movement patterns, variability around the characteristic scales is huge and statistically self-similar such that variability across a range of scales resembles variability occurring across all scales. This is quite unlike multiphasic (e.g. bimodal) search patterns which typically combine just two scales of searching, intensive (within-patch) searching and extensive (for patch) searching. The Lévy search strategy, hereafter called a ‘Lévy flight pattern’, is also evident in honeybees when they are artificially displaced to an unfamiliar location and searching for the hive^14^ or when searching after a known food source that has been removed^15^. The implementation of this search strategy does not necessarily require sophisticated cognitive abilities on the bee’s part, as Lévy flight patterns can be derived from the Weber–Fechner law in a bee’s odometer, where errors in the estimates for the distance flown increase linearly with the actual distance flown^16^. In this case, the flight patterns stem from the observed proportionate growth in loop lengths, which can be attributed to bees attempting unsuccessfully to reproduce loop lengths because of errors in distance estimation^16^.

The general characteristics of these two-dimensional looping flights can be used to predict how bees will find new resources in more complex and ecologically realistic three-dimensional landscapes (where food resources are at different heights relative to the ground). In the presence of a single target, the stereotypical search strategy begins at the location where the forager initially expects to find the target, and is comprised of loops of increasing size that start and end at this location, and are directed in different azimuthal directions^14^. This ends when the target of the search is found and is otherwise curtailed by biological or environmental constraints. We hypothesised that this is manifestation of a ‘simulated annealing’ strategy, a powerful stochastic search algorithm for locating a global maximum (best resource) that is hidden among many poorer local maxima (poorer resources) in a search space, inspired by a heat treatment procedure in metallurgy^17^,^18^,^19^. This possibility has not been examined before but if a forager were to use this strategy when attempting to locate high quality food resources among many poorer resource patches then it would begin as described above with looping flights centred on a found food location. This search pattern would continue until a new food location is found. If the new food is more profitable than the previous one then the search pattern would become centred on the location of the newly found food. If the new food is worse than the previous one the search patterns would become re-centred on the previous food location. The cycle would then be repeated many times and at each step the probability of accepting worse food locations would decrease (‘cool’) as the forager explores its environments. Accepting worse solutions is a fundamental property of simulated annealing because it allows for a more extensive search for the best target^17^. The efficiency of the heuristic can be increased by occasional ‘re-heating’ during which the iteration count is reset. Through this process the searcher can quickly escape from local maxima. Simulated annealing is most effective when targets are hard to locate, for instance when they are distributed in three-dimensions.

Simulated annealing has been applied to numerous optimization problems in engineering and in the physical sciences^20^. The first applications used so-called ‘Boltzmann’ annealing which is based on strictly local sampling, as step-lengths in the search patterns are Gaussian or exponentially distributed with a single characteristic scale. A major drawback with Boltzmann annealing is its slow rate of convergence to the optimal location. The problem was overcome by Szu and Hartley^17^ who introduced ‘fast simulated annealing’ in which searching patterns are like Lévy flights, i.e. step-lengths are drawn at random from a distribution with a heavy power-law tail. This search algorithm does, in fact, outperform all Boltzmann-like simulated annealing schemes, i.e., all schemes that utilize thin-tailed step-length distributions^17^. Thus, in principle, fast simulated annealing search patterns could enable animals foraging for cryptic resources, such as bees searching for floral nectar, to quickly locate the best available resource patches in a complex environment containing many alternatives of lower qualities. Since this process involves that looping movements will be re-set and re-centered each time a better location is found, simulated annealing search cannot be observed when searching in the presence of only a single target or in the absence of any targets, as was the case in previous studies in bumblebees^12^ and honeybees^14^,^15^.

Here we examined the extent to which the three-dimensional searching flights of foraging bumblebees are consistent with their utilizing fast simulated annealing strategy in a situation where bees had to search for artificial flowers in an enclosed flight arena. To simplify the analysis we focused exclusively on visual search and therefore excluded all olfactory cues that could flood the entire arena, making their influence on search behaviour difficult to discern. Individual bees were tested over multiple foraging bouts between which the three-dimensional spatial configuration of flowers was randomly changed. To assess whether search patterns vary in environments where targets are more or less difficult to locate, the same bees were tested with large and small flowers thereby reducing flower detectability. The resulting movement data were compared with well-established alternative search models: ‘slow’ Boltzmann-like simulated annealing, fast simulated annealing and bi-modal searching.

## Methods

### Flight arena and flowers

**Figure 1:**
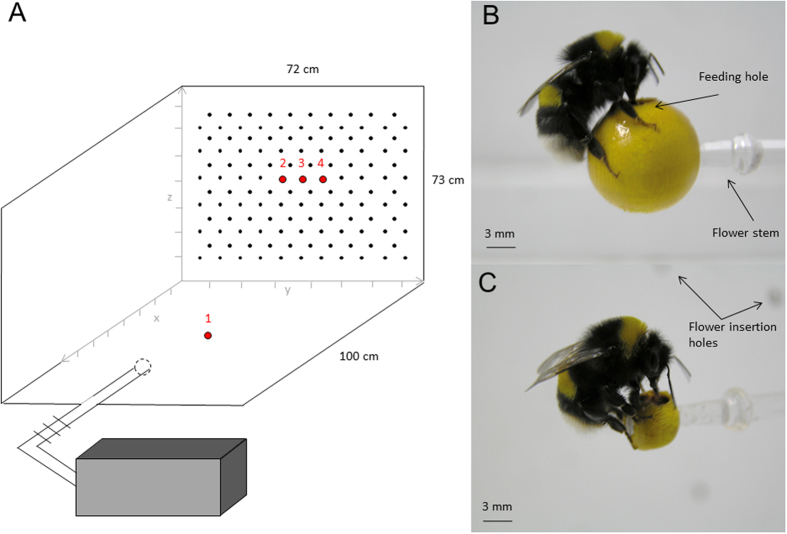
Experimental setup. (**A**) The colony nest box is connected to the flight arena via a transparent entrance tube fitted with shutters. The observation wall in the background is covered of a grid of 126 holes in which the stems of artificial flowers are inserted. Prior to the experiments, bees were trained to forage on a single flower (location 1) in the middle of the arena. Regular foragers were further allowed to collect sucrose solution from three flowers attached to the observation wall (locations 2, 3 and 4) for three foraging bouts. During the tests, bees were presented three flowers attached to the observation wall by glass stems of varied length (1–30 cm) during six consecutive foraging bouts (bouts 1–3: large flowers, bouts 4–6: small flowers). The spatial configuration of flowers on the wall was changed between each bout. Three-dimensional coordinates of bees were recorded with high-speed video cameras in the area delimited by the grey x-, y- and z-axes. (**B**) Photo of a bumblebee forager (*Bombus terrestris*) collecting sucrose solution through a feeding hole in the centre of a large flower (Ø: 12 mm). (**C**) Bumblebee on a small flower (Ø: 6 mm). Photographs by ML.

All experiments were performed with individually marked bumblebee workers from a commercially obtained *Bombus terrestris* colony (Syngenta Bioline Bees, Weert, The Netherlands). The colony nest box was connected to a wooden flight arena (l = 100 cm, w = 72 cm, h = 73 cm, v = 525 600 cm^3^) with a transparent plastic tube fitted and a series of shutters to control bee traffic (Fig. 1A). The floor and inner walls of the arena were coated with white paint (Leyland, Bristol, UK). The arena was covered with a UV-transmitting Plexiglas lid. The colony was kept at room temperature (ca. 23 °C) on a 12:12 h L:D cycle (light on at 08:00 hours) throughout the experiment. Controlled illumination was provided by high frequency fluorescent lighting [TMS 24F lamps with HF-B 236 TLD (4.3 KHz) ballasts, Phillips, Eindhoven, The Netherlands] fitted with Activa daylight fluorescent tubes (Osram München, Germany), emitting light with a flicker (4.3 KHz) well above the flicker-fusion frequency of bees^21^ and mimicking natural daylight, including a near-ultraviolet component^22^. The colony was provided with *ad libitum* defrosted pollen (Koppert BV, Berkel en Rodenrijs, The Netherlands) directly into the nest.

Workers collected sucrose solution (40% w/w) from artificial flowers attached to one wall (observation wall) of the arena (Fig. 1A). Flowers were made of yellow wooden beads mounted on transparent colourless glass rod stems (l: 300 mm, Ø: 2 mm). We used large flowers made of a 12 mm Ø beads (Fig. 1B) and small flowers made of 6 mm Ø diameter beads (Fig. 1C). The colourless stems were inserted into holes in the observation wall of the arena and pushed through to varying depths, thereby creating a three-dimensional array in which the flowers protruded to different distances from the wall (x-dimension, range: 0–30 cm), and at different heights (z-dimension, range: 3–70 cm) across the entire wall width (y-dimension, range: 2.5–70.5 cm). Thus, the flowers were distributed in three dimensions, as it is the case for many natural meadows (where flowers vary in height). During the tests, flowers were randomly arranged on an orthogonal grid of 126 holes (distance between nearest neighbour holes: 56 mm) using the function ‘sample’ in the *base* R package^23^ to draw uniformly-distributed random numbers. Hole locations were randomly distributed between 1 and 126. Stem lengths were randomly distributed between 1 and 30 (step size = 1 cm). The same randomisation procedure was used to generate arrays of large flowers and small flowers. Bees could land on flowers and access a pre-set volume of sucrose solution through a feeding hole in the middle (Ø: 1 mm, maximum capacity: 50 μL; Fig. 1B,C). The artificial flowers and sucrose solution were not scented. Therefore, bees could only locate them using vision. Since *B. terrestris* workers are able to detect reflecting (non self-luminant) visual targets from a background subtending a visual angle of ca. 3° 24, we assume that bees could detect a large flower from a maximal distance of ~23 cm and a small flower at ~11.5 cm from any location in the arena. Testing bees in experimental setups whose dimensions are larger than the visual detection range of flowers induces search behaviour. This approach has been successfully adopted in numerous previous studies investigating bees’ search strategies in two-dimensional environments^11^,^25^,^26^,^27^,^28^.

### Experimental procedure

Bees were pre-trained to forage *ad libitum* on a large stemless training flower (Ø: 12 mm) placed on the floor in the centre of the arena (location 1, Fig. 1A). This procedure, during which multiple individuals could enter the arena and forage simultaneously, enabled bees to familiarise themselves with the flower design and the flight arena. Regular foragers that made at least five foraging bouts in one hour (collected sucrose solution from the flower until they filled their crop to capacity and returned to the nest box) were selected for testing. These bees were then allowed to forage sequentially on three large training flowers placed in the middle of the observation wall (locations 2, 3 and 4, Fig. 1A) for another three foraging bouts to make them learn to search for flowers in the three-dimensional space. The average volume of sucrose solution ingested by each bee during these foraging bouts was used to estimate their individual crop capacity (range: 70–150 μL)^28^.

Observations were conducted in two phases. In phase 1, a bee was tested for three consecutive foraging bouts (bouts 1–3) with the three large flowers presented in the three dimensional space. Each flower contained 1/3 of the bee’s crop capacity. Flowers were not refilled with sucrose solution following a bee visit during a continued foraging bout, thereby inciting bees to find the three flowers in order to fill their crop to capacity^28^. Between bouts (when bees had returned to the nest box), we changed flowers to avoid the potential influence of chemical scents deposited on flowers during previous visits on search behaviour^29^. We also randomly changed the spatial arrangement of flowers so that bees discovered a new foraging situation in each bout (see randomisation procedure above). In phase 2, we repeated the same procedure with three small flowers (bouts 4–6) thereby making the now familiar task more difficult (due to the 50% reduced size of flowers). This phase was designed to explore the effect of a reduction of flower detectability on search behaviour. A total of 15 bees (age 10–15 days post eclosion from the pupae) were used. Only one bee was tested at a time (ca. 2 h of continuous observations per bee).

#### Data collection

The flight behaviour and position of bees were recorded with video tracking. Three-dimensional positions of bees were calculated 50 times per second with two video cameras connected to a computer running Trackit 3D (cameras and software from BIOBSERVE GmbH, Bonn, Germany)^27^. The tracking system covered 3/4 of the volume of the arena (including the observation wall) where the search activity of bees was the most important (as delimited by the grey axes on Fig. 1A). A third camera (Sony camcorder HDR-AX2000), positioned above the arena, was used to record videos of the entire arena surface in order to reconstruct the behaviour of bees during complete foraging bouts, from the moment when the bee leaves the colony nest box to its return.

#### Analysis of search performances

We extracted information about the complete flight sequences of individual bees from the actual video data (not the three-dimensional coordinates) collected in parallel to the tracking system. For each foraging bout of each bee, we reconstructed the flower visitation sequence (starting and ending at the nest box), and computed the number of immediate revisits to flowers (when bees returned to a flower immediately after having visited that flower within the same foraging bout) and the number of delayed revisits (when bees returned to a flower after having visited at least one different flower in between within the same foraging bout). We also calculated the duration of foraging bouts (total time spent between the moment when the bee leaves the colony nest box to its return), the total time spent flying per foraging bout, as well as the latencies to discover the 1^st^, 2^nd^ and 3^rd^ flowers per foraging bout (time of the first visit to each flower). Comparing these data for observational phase 1 (bouts 1–3) and phase 2 (bouts 4–6) yielded information about how search performances are affected by manipulations of flower size.

#### Analysis of flight patterns

We extracted information about flight patterns from the three-dimensional coordinates collected by the tracking system. To analyse flight steps (bouts of near unidirectional flight), the three-dimensional flight patterns were first projected on the x-, y- and z-axes, creating projections for each recorded flight pattern. Turns in the projected flight patterns can be identified in an unambiguous way as occurring when the direction of travel changes. Without projection, turns can only be identified by making reference to arbitrarily defined critical-turning angles^30^. Humphries *et al*.^30^ showed that projection does not affect the distribution of the distances travelled between consecutive turns. This projection method was recently appraised and adapted by Tromer *et al*.^31^. Because the bees typically flew back and forth the same flower many times, most flight steps can be regarded as being flight ‘loops’. Following Edwards *et al*.^32^, model distributions were fitted to our data using maximum likelihood methods, and the best model distribution was identified the Akaike information criterion. These methods have now become standard to compare the outputs of competing models in the literature on Lévy flights. Using the Akaike information criterion^33^, we tested whether the distributions of the bees’ flight-step lengths *l* provided more evidence to have a power-law tail (fast simulated annealing model)


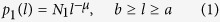


a bi-exponential tail (bi-modal searching model)


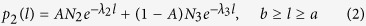


or a single exponential tail (Boltzmann-like simulated annealing model)


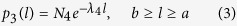


where *N*_*1*_, *N*_*2*_, *N*_*3*_, and *N*_*4*_ are normalization factors which ensure that the probability distributions, *p*_*1*_, *p*_*2*_, and *p*_*3*_, sum correctly to unity when integrated over all flight-step lengths between *a* and *b*. A power-law distribution of flight-step lengths is indicative of Lévy flight patterns and fast simulated annealing. A bi-exponential tail could be indicative of bees switching between two modes; extensive searching for flowers and intensive searching of found flowers. Bi-exponentials can closely resemble power-laws when, as in the current situation, the range of scales is limited, and so can compete strongly with Lévy flights as models of movement pattern data^34^. A single exponential tail, together with looping back-and-forth around found flowers is indicative of ‘slow’ Boltzmann-like simulated annealing^17^. Without looping they are indicative of ‘correlated random walks’, which provide the dominant conceptual framework for the modelling of movement pattern data^35^. The power-law exponent, *μ*, the relative weights of the two exponentials in the bi-exponential, *A* and *1-A*, and the exponential decay rates, *λ*_*2*_, *λ*_*3*_, *λ*_*4*_, were determined using log-maximum likelihood methods^36^. The start of the tail of the distributions (*a* ≈ 0.01 *m*) was ascertained by visual inspection of the survival function (the complement of the cumulative distribution function). The upper bound, *b*, was taken to be the longest recorded flight step. We also compared our data with a modified form of power-law distribution that takes explicit account of flight-step truncation at the boundaries of the flight box and at found targets^37^. To construct the survival function, the simulation data for the distances {*l*_*i*_} was first ranked from largest to smallest {*i* = 1…*n*}. The probability that a length is greater than or equal to *l*_*i*_ (the survival function) is then estimated as *i*/*n*. Flight steps containing gaps where recordings are missing were not included in the analysis. We also excluded from the analysis flight steps where a centroid of tracked pixels was incorrect and resulted in flight speeds > 5 m/s (18 km/h) which the bees are incapable of within such a small flight arena.

The absolute goodness of fits of the model distributions were quantified following an approach advocated by Clauset *et al*.^36^ which furnishes a p-value. An observed distribution is extremely unlikely to follow a power-law or exponential form exactly as there will always be some small deviations because of the random nature of the sampling process. The approach of Clauset *et al*.^36^ distinguishes deviations of this type from those that arise because the data are in actuality not power-law distributed. If p is large (p > 0.1), the difference between the empirical data and the model can be attributed to statistical fluctuations alone; if it is small (p < 0.1), the model is not a plausible fit to the data. As noted by Clauset *et al*.^36^ the more lenient rule p ≤ 0.5 is type 1 error prone.

Flight speeds were determined from the distances travelled between consecutive recordings. To test for the presence of correlations of movement in different dimensions we identified vertical displacements (with speeds ≥0.5 m/s and <5 m/s), found the accompanying horizontal movement and then counted the numbers of vertical displacements in each horizontal speed bin of width 0.05 m/s. In this way we can determine whether or not vertical displacements tend to be accompanied by horizontal movements. An analogous calculation was performed for horizontal displacements (with speeds ≥0.5 m/s and <5 m/s).

To estimate flower detection range by bees from the three-dimensional coordinates, we identified the occasions when a bee first came within 1 cm of a particular flower (minimum distance at which bees always get in contact with a flower). We then worked backward along the track and determined the location at which the flight heading was first on-target i.e., location after which all subsequent headings, if maintained, would bring the bee to within 5 cm of the flower (this 5 cm circle is used essentially as a measure to quantify path straightness) (Figures S1 and S2 in the Supplementary Material). We took the distance between this location and the target to be the detection range. This approach is robust as outcomes do not change significantly when 5 cm is replaced by values either 50% smaller or 50% larger) but will occasionally overestimate the detection range, as some bees may be on the right heading by chance. The 5 cm threshold is less than actual estimations of flower detection range based on spatial resolution of the bee vision system (i.e. ~23 cm for large flowers, ~11.5 cm for small flowers, given that *B. terrestris* workers can detect non-self-luminant targets that subtend a minimum of ca. 3^o^ 24). However, it provides a quantitative measure for comparing search patterns among different bees and between the two phases of the experiment.

#### Statistical analyses

All statistical analyses were performed in R v 3.1.2^23^. Measures of search performances in the presence of large and small flowers were compared with Generalised Linear Mixed Models (GLMM) using the function ‘glmmPQL’ (R package *MASS*^38^). In all models we included flower size (large, small) as fixed effect and subject (bee identity) as random factor to account for repeated measures. We selected appropriate family error structure (Gaussian, Poisson or Gamma) depending on the distribution of the error terms. Means are given with their standard error (mean ± s.e.).

## Results

### Search performances

First we examined the overall search performances of bees in the presence of large flowers and small flowers. These analyses are based on the actual video data (not the three-dimensional coordinates) of 11 individuals (videos for bees w50, w54, w55 and w66 were not complete). We found that the duration of foraging bouts increased by 50% with decreasing flower size (Table 1). Accordingly, the total time spent flying per foraging bout was two times longer with the small flowers (Table 1). The latencies to discover the 1^st^ flower, the 2^nd^ flower and the 3^rd^ flower also tended to increase (e.g. by 95% for the 1^st^ flower) with the small flowers (Table 1). Flower visitation sequences (including all visits to flowers during a foraging bout) were consistently longer with the small flowers (Table 1). This difference is explained by a 65% increase of the number of immediate revisits to empty flowers per foraging bout with small flowers (Table 1). However, there was no difference in the frequency of delayed revisits to flowers per foraging bout, i.e. when bees returned to a flower after having visited at least one different flower in between (Table 1). Presumably, the shorter visual detection range combined to the greater frequency of flight loops between immediate revisits to flowers caused bees to fly for longer before finding small flowers than large flowers, thereby producing a significant increase of search times with decreasing flower size.

### Structure of flight patterns

**Figure 2:**
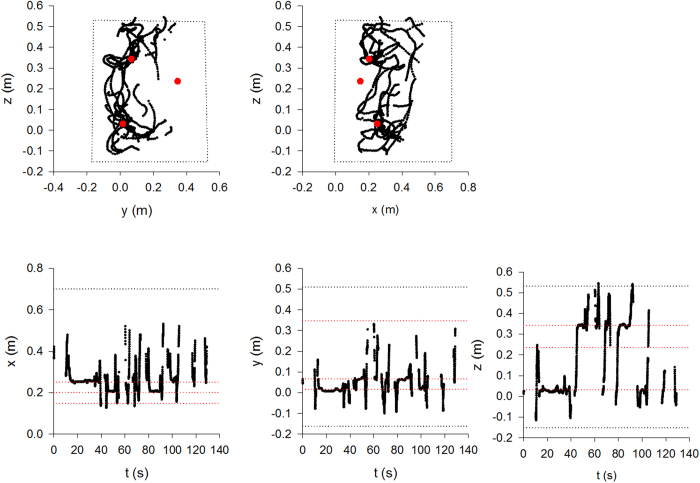
Complete recording of a flight pattern showing looping characteristics indicative of fast simulated annealing together with the time courses of the x-, y- and z-position coordinates. The example flight pattern (bee b66 on bout 1) (●) has been projected onto the y-z and x-z planes. The flight patterns are confined by the borders of the flight arena (black dotted lines) and take place in the presence of three large flowers (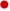, red dashed-lines). In this example only two flowers are found.

**Figure 3:**
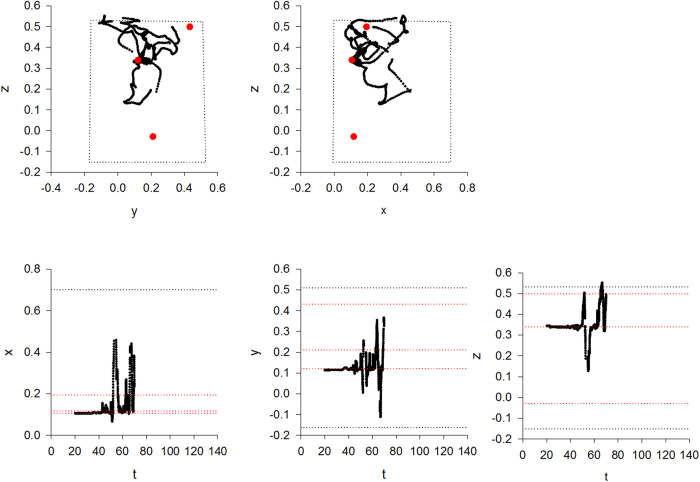
Partial recording of a flight pattern showing details of looping characteristics indicative of fast simulated annealing together with the time courses of the x-, y- and z-position coordinates. This looping is not evident when the entire flight pattern is plotted because it fills most of the space. The example flight pattern (bee b66 on bout 4) (●) has been projected onto the y-z and x-z planes. The flight patterns are confined by the borders of the flight arena (black dotted lines) and take place in the presence of three large flowers (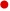, red dashed-lines). In this example only one flower is found.

**Figure 4:**
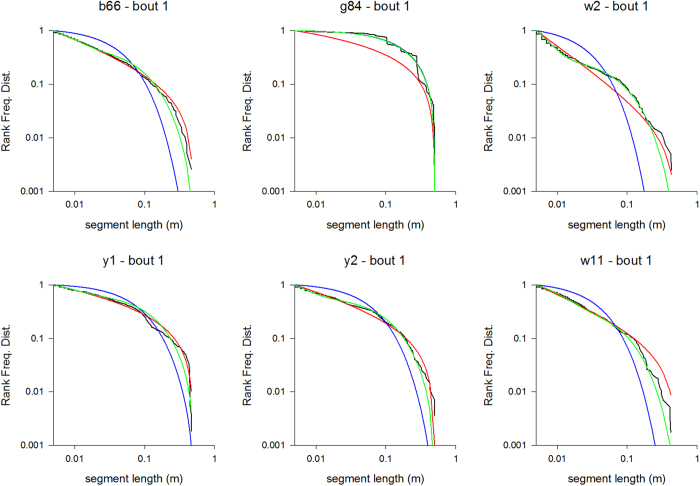
Rank frequency distributions of flight-step lengths tend to show power-law scaling indicative of fast simulated annealing. Rank frequency distributions for bees b66, g84, w2, y1, y2 and w11 made during their first foraging bouts and in the presence of large flowers (black lines), and shown together with the best fit power laws (red lines), the best fit bi-exponentials (green lines) and the best fit exponentials (blue lines). Data have been pooled for the x-, y- and z-directions. The numbers of flight-steps are given in Table 2.

**Figure 5:**
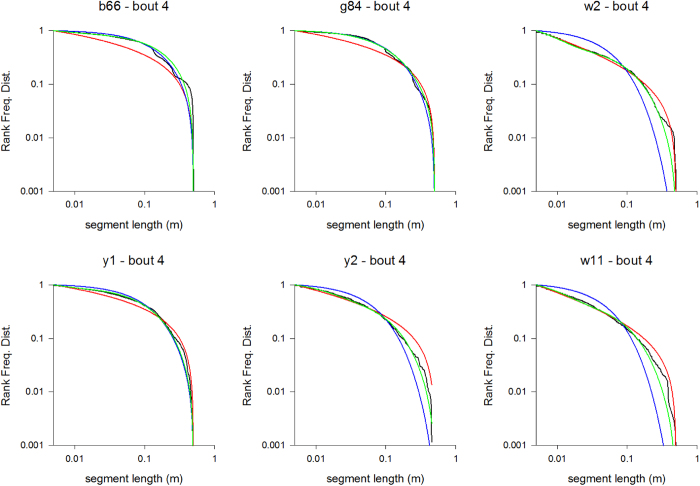
Rank frequency distributions of flight-step lengths tend to show looping power-law scaling indicative of fast simulated annealing. Rank frequency distributions for bees b66, g84, w2, y1, y2 and w11 made during their fourth foraging bouts and in the presence of small flowers (black lines), and shown together with the best fit power laws (red lines), the best fit bi-exponentials (green lines) and the best fit exponentials (blue lines). Data has been pooled for the x-, y- and z-directions. The numbers of flight-steps are given in Table 3.

To further examine these differences of search performances in the presence of large and small flowers, we analysed the fine structure of the flight patterns of the 15 bees using their three-dimensional tracking coordinates. From the complete dataset we discounted aberrant tracks containing erratic data which contained abnormally large numbers (>1000) of flight-steps. Our quantitative analyses of flight patterns revealed that bees tend to move in loops of ever increasing size that are centered on a flower (Figure S3). The loop size is reset whenever another flower is encountered, and the looping is re-centered around that flower (see examples in Figs 2, 3 and S4). This gradual increase in loop length is observed irrespective of the flower’s three-dimensional location, indicating that looping is not simply triggered by the presence of the arena walls. Typically flight-step lengths do not follow exponential distributions (Tables 2, 3 and S1–S4). Thus bees’ flight searches are generally not compatible with either exponential (Boltzmann-like simulated annealing model) and bi-exponential (bi-modal searching model) flights patterns. The flight patterns of 11 out of the 14 bees on their first foraging bouts (bout 1) in the presence of large flowers displayed evidence of power-law scaling (fast simulated annealing model), i.e., the Akaike weights for power-law distributions were >0.3 (see examples in Fig. 4, Table 2). The maximum likelihood estimates for the power-law exponent ranged between 1.14 and 2.36. Power-law scaling is evident for flight-steps ranging between about 1 cm and about 30 cm which is about the average distance to the walls where flight steps must be truncated. Note that truncated power-laws are curvilinear when plotted on log-log scales^39^. Analogous results were obtained for bouts 2 and 3 which were also obtained in the presence of large flowers (Tables S1 and S2), and when the power-law distribution was modified to take explicit account of flight-step truncation (analysis outcomes not shown). In bout 2, 9 out of 14 flights displayed evidence of power-law scaling and in bout 3 this scaling was evident in 12 out of 14 flights. The good fits to a power-law distribution is a clear signature of Lévy flight patterns, validating the fast simulated annealing hypothesis and discarding the Boltzmann-like simulated annealing and bi-modal searching models. For bouts 4–6 made in the presence of small flowers, power-law scaling was evident in 6 out of 8 flights (see examples in Fig. 5, Table 3, Tables S3 and S4), 4 out of 10, and 4 out of 11 flights respectively. Reducing flower size is seen to reduce the prevalence of power-law scaling and to increase the number of aberrant flight patterns (presumably because flower movement, consecutive to a bee visit or when a bee approaches a flower, is creating noisy pixels and because this is worse for small flowers). Such noise in the data could occasionally lead to bi-exponentials appearing to be the better model.

**Figure 6:**
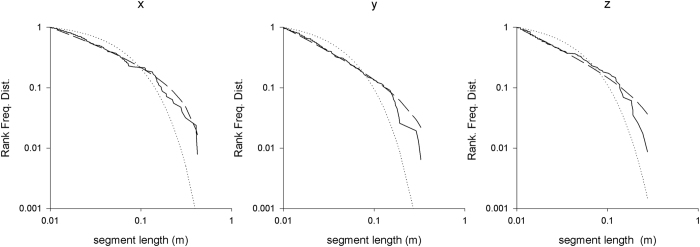
Rank frequency distributions of flight-step lengths tend to show near isotropic looping power-law scaling indicative of fast simulated annealing. Rank frequency distributions for bee w11 made during its first foraging bout and in the presence of large flowers (solid-lines), and shown together with the best fit power laws (dashed-lines) and best fit exponentials (dotted-lines). The Akaike weights for power-law scaling being the better scaling are all 1.00 and the maximum likelihood estimates for the power-law exponents are 1.46, 1.73 and 1.69 for the x-, y- and z-directions. The numbers of flight-steps are 126 (x), 155 (y) and 115 (z).

**Figure 7:**
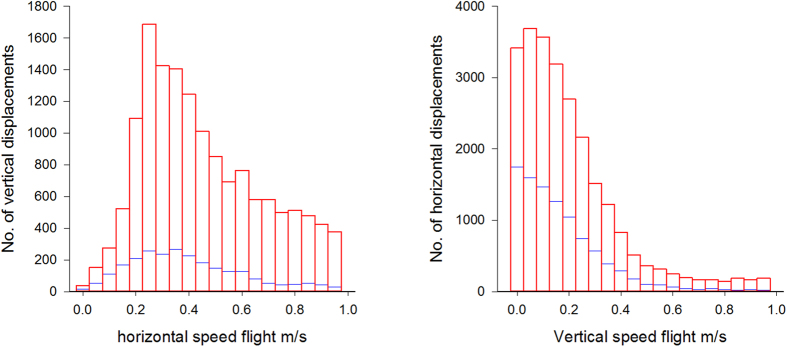
Vertical displacements (with speeds ≥ 0.5 m/s) are accompanied by horizontal displacements (left panel). Horizontal displacements (with speeds ≥ 0.5 m/s) need not be combined with vertical movements (right panel). Data have been pooled for all flights made in the presence of the large (blue bars) and small (red bars) targets (15 individual bees each making 3 flights).

There is no indication that the bees’ looping strategy differs with their orientation in the three-dimensional space. Looping can be equally pronounced in all three dimensions and flight-step lengths show near isotropic looping power-law scaling (see example in Fig. 6). Nonetheless, upwards movements were always accompanied by horizontal movements (resulting in diagonally upward flights), whereas horizontal movements did not need to be combined by vertical movements, an ‘accompanying effect’ evident in our data (Fig. 7). Presumably, bees can fly straight horizontally but not straight up because in flight their body axis is typically kept horizontal^40^,^41^ and forward flight is used to adjust the plane of wing vibrations during upward or downward flight^42^. Average flight speeds per individual were similar in the presence of both the large and the small flowers (Table 1). However, as expected from the visual perception system of bees, our estimations of detection ranges were shorter for the smaller flowers [large flowers: 7.79 ± 0.44 cm (maximums range 9.2–14.8 cm, N = 24), small flowers: 6.97 ± 0.33 cm (maximums range 4.2–12.5 cm, N = 24), GLMM with Gaussian error structure and subject as random factor, t_48_ = 3.03, p = 0.0039]. This suggests that, the observed differences in search times in the presence of large and small flowers are not driven by changes of search strategies but simply by differences in flower visual detection range.

## Discussion

We examined the flight patterns of bumblebees searching for floral targets of various sizes randomly arranged in a three-dimensional space. In contrast to previous observations that bees search systematically with relatively directed flights when flowers are evenly spaced with predictable locations^26^, our results show that foragers use a more complex probabilistic searching strategy when flowers are randomly distributed in space and difficult to locate. After finding a flower, a bee tends to scan back-and forth in the vicinity of that flower for a time before prospecting away from that flower, and if not finding anything returning to the original flower. The bee subsequently makes more prospecting flights (loops) and the length of these flights tend to increase over time (eventually causing some bees to encounter the walls of the arena). The looping pattern becomes gradually more expansive and culminates when another flower is found. This search behaviour, whose flight-step lengths are typically power-law (heavy-tailed) distributed is reminiscent of fast simulated annealing^17^. The Akaike information criterion usually favours power-law fits (fast simulated annealing) over exponential (Boltzmann-like simulated annealing) and bi-exponential (bi-modal search) fits, and in most cases the power-laws provide good fits to our flight-step length data (see Akaike weights and p-values in Tables 2, 3, S1, S2 and S3).

To our knowledge, fast simulated annealing search has not been described previously in nature. Many animals, such as ants^43^,^44^, honeybees^14^,^15^ and isopods^45^, are known to exhibit search loops of ever-increasing sizes when attempting to relocate a familiar target such as their nest, but there is no evidence that these behaviours are used by foragers to explore and locate novel resources. This kind of Lévy searching strategy that involves loops back-and-forth from a central point also differs significantly from the freely roaming Lévy searching strategy that can be advantageous when searching for any target rather than the best available target^46^,^47^, as observed to some extent in bacteria^48^, T cells^49^, a diverse range of marine predator^39^,^50^, mussels^51^, mud snails^52^, the wandering albatross^30^, extinct marine organisms^53^ and human hunter-gatherers^54^. Nonetheless, there is some evidence that the unusually large jellyfish *Rhizostoma octopus* occasionally search the water column for the prey rich strata using a strategy resembling fast simulated annealing^55^. The movement patterns of this jellyfish are consistent with individuals prospecting away from a preferred depth, not finding an improvement in conditions elsewhere and so returning to their original depth. Excursion characteristics vary over time and in many instances exhibit power-law scaling indicative of ‘Lévy looping’^56^.

Importantly, we show that bumblebees consistently used this search strategy across successive foraging bouts and in the presence of flowers of different sizes. Reducing flower size increased overall search time and decreased the performance of bees in locating flowers. This result is consistent with previous studies in which the manipulation of the optical properties of flowers (size or colour) affected search time of foraging bumblebees because of inherent visual constraints^11^. However, our analyses did not detect changes in flight behaviour or flight speed in the presence of large and small targets. While foraging in strictly horizontal arrays (where all flowers are presented level with the arena floor) forced bees to perform search flights close to the ground and thus to slow down^11^, foraging in the three-dimensional space did not induce more search close to the floor or to the walls of the arena, which may explain why we did not observe a general slowing down effect in the presence of small flowers in this new study. Rather our data suggest that increased search time is only a consequence of the lower probability to detect a small target using random looping search due to shorter detection range. Behavioural estimates for the maximum detection range (~14 cm for large flowers and ~7 cm for small flowers) are broadly consistent with the expectation that objects first become visually detectable by a bumblebee worker when they subtend about 3 degrees^24^. Since flowers were free of chemical marking (i.e. unscented and replaced between every foraging bouts), bees could only locate them by vision. Low volatile scent marks (cuticular hydrocarbons) deposited by bees through successive visits on flowers could potentially be used to relocate previously discovered flowers over short distances^29^, but these revisits were not rewarded.

Analyses of flight structures also revealed potentially important features of the search efficiency by bees based on their differential ability to move straight when flying horizontally or vertically. Horizontal search patterns (which do not need to be accompanied by vertical displacements) enable individuals to cover larger surfaces and may therefore increase search efficiency in two-dimensional spaces. Conversely, being forced to move in both horizontal and vertical directions when flying up and down (the ‘accompanying effect’) increases the chances of covering a larger volume with random looping movements, which may also increase the probability of finding hidden targets in a three-dimensional space. Thus in theory, bees’ flight patterns may yield different search efficiencies in environments where flowers are primarily arranged horizontally, vertically or in both planes. This is consistent with recent observations that the foraging performances of bumblebees are dependent on whether flowers are presented horizontally or vertically^57^ or whether foragers approach flowers from above or from below^58^. While our study only involved analyses of search behaviour in a relatively confined space at small spatial scales (100 × 72 × 73 cm arena), there is no reason why these strategies would not be expressed in open spaces and/or at larger spatial scales, in natural conditions. First, the observation that the looping pattern becomes gradually more expansive as bees search for flowers indicates that flight loops are not a direct consequence of boundaries. If bees were simply attempting to avoid collisions with walls, we would expect loops centred at a given flower to have constant amplitude throughout the search process, at a stable distance from the walls. Second, the observation that flower size limits flower detectability is consistent with knowledge on the relatively poor visual-spatial resolution of the bee eye^58^,^59^ and numerous previous studies of two-dimensional search behaviour by bees in the lab^11^,^27^,^28^ and the field^25^. Whether and how the presence of additional floral signals, such as the volatile odours emitted by natural flowers^60^, influence these search patterns in the field remain to be tested.

The possibility that bumblebees use fast simulated annealing search for locating best resources in their patchy and dynamic foraging environments is consistent with recent analyses of their flight patterns at larger spatial scales^25^ and brings new insight into how bees may learn to exploit arrays of flowers from a central nest, using multi-location routes (traplines) between most profitable feeding locations (inflorescences, flower patches or plants). Upon finding a flower, bumblebees make looping flights away from that flower, as though they are searching for other flowers, eventually locating all available resources within a patch^25^. In this process, fast simulated annealing search may initially improve the ability of foragers to find and learn the spatial location of best rewarding feeding locations, thereby providing the basis for a route to develop. Through repeated trials, bees may then gradually refine their route by re-arranging the order in which they visit selected locations while continuing exploring for new (more rewarding) locations to incorporate^61^. Ultimately, the combination of exploration (as per fast simulated annealing search) and exploitation (iterative improvement of route efficiency) may lead to route optimisation, whereby bees prioritize visits to most rewarding locations^62^ while reducing travel distances between them^28^,^63^,^64^. When multiple bees forage in the same area, the employment of simulated annealing search to locate resources and establish foraging routes may also facilitate space partitioning^65^ and the emergence of ‘ideal-free distributions’^66^ whereby foragers self-distribute on floral resources in such a way that energy gains are equalized across all foragers^67^. Specifically, the searching patterns we describe would enable foragers to move (or tend to move) in the direction of increasing feeding site quality, a local dispersal rule resulting in trapping different individuals in different local maxima.

Fast simulated annealing search is a relatively simple yet potentially highly efficient strategy for a wide range of animals foraging on ephemeral floral resources to find high-reward patches among multiple alternatives of various nutritional qualities, such as nectar feeding insects, birds, reptiles and mammals. Thanks to the development of high-resolution technologies for tracking and analysing animal movements^68^,^69^, it is now becoming possible to start exploring how widespread this strategy is by comparing fine scale search patterns across taxonomic groups and ecological contexts within an evolutionary framework. Future approaches implementing pollinator search and learning algorithms into simulation models (e.g. ref. 61) and autonomous robots (e.g. ref. 70) will help identify to what extent such computationally simple search strategies may be effective across different foraging environments and understand how they may shape the complex spatio-temporal distributions of flower foragers in real landscapes.

## Additional Information

**How to cite this article**: Lihoreau, M. *et al*. Signatures of a globally optimal searching strategy in the three-dimensional foraging flights of bumblebees. *Sci. Rep.* **6**, 30401; doi: 10.1038/srep30401 (2016).

## Supplementary Material

Supplementary Information

## Figures and Tables

**Table 1:** Summary statistics for the search performance of bees in the presence of large and small flowers.

Behavioural measures	Large flowers	Small flowers	t	df	p
Bout duration (s)	282.25 ± 21.69	421.87 ± 37.98	3.90	48	0.0003
Time spent flying (s)	100.73 ± 9.51	233.67 ± 29.99	4.97	48	<0.0001
Latency to discover 1^st^ flower (s)	16.03 ± 1.80	31.30 ± 6.46	3.66	48	0.0006
Latency to discover 2^nd^ flower (s)	100.65 ± 9.63	134.30 ± 17.08	2.80	46	0.0075
Latency to discover 3^rd^ flower (s)	193.54 ± 18.62	317.74 ± 60.30	3.21	32	0.0030
No. flower visits	11.82 ± 1.38	17.89 ± 1.65	3.03	48	0.0039
No. immediate revisits	6.33 ± 1.06	10.47 ± 1.19	3.05	48	0.0037
No. delayed revisits	2.74 ± 0.53	4.79 ± 1.14	1.81	48	0.0773
Flight speed (m/s)	0.15 ± 0. 01	0.27 ± 0.01	1.36	74	0.1780

Means are showed with their standard errors (mean ± s.e.). p-values indicate the results of Generalised Linear Mixed Models (GLMM) with subject (bee identity) as random factor.

**Table 2:** Summary statistics for the structure of flight patterns of bees on their first foraging bouts in the presence of large flowers (bout 1).

Bee	Akaike weights for a power-law, a bi-exponential and a single exponential	Power-law exponent μ	No. of flight-steps	p-value for the power-law
b66	1.00, 0.00, 0.00	1.48	386	1.00
g84	—	—	3072	—*
y1	1.00, 0.00, 0.00	1.15	538	0.00
y2	0.93, 0.07, 0.00	1.34	276	0.38
w2	0.00, 1.00, 0.00	1.95	402	—
w11	0.98, 0.02, 0.00	1.54	567	1.00
w20	0.01, 0.99, 0.00	1.30	440	0.00
w49	0.41, 0.59, 0.00	1.41	255	0.07
w50	1.00, 0.00, 0.00	2.36	1476	1.00
w54	0.97, 0.03, 0.00	1.31	302	0.62
w55	1.00, 0.00, 0.00	1.78	334	0.98
w57	0.40, 0.60, 0.00	1.83	459	0.99
w67	0.98, 0.02, 0.00	1.57	328	0.99
y80	0.51, 0.49, 0.00	1.54	178	0.57
y81	0.00, 1.00, 0.00	1.14	363	—

p-values quantifying the goodness-of-fit of the power law distributions to the data. * denotes noisy erratic recordings.

**Table 3:** Summary statistics for the structure of flight patterns of bees on their first foraging bouts in the presence of small flowers (bout 4).

Bee	Akaike weights for a power-law, a bi-exponential and a single exponential	Power-law exponent μ	No. of flight-steps	p-value for the power-law
b66	0.00, 1.00, 0.00	—	2918	*
g84	0.00, 0.99, 0.01	—	2456	*
y1	0.00, 1.00, 0.00	—	1690	*
y2	0.00, 1.00, 0.00	1.20	861	—
w2	0.01, 0.99, 0.00	1.38	1024	
w11	1.00, 0.00, 0.00	1.40	1084	1.00
w20	0.00, 1.00, 0.00	—	2261	—*
w49	0.00, 1.00, 0.00	—	4340	—*
w50	0.05, 0.95, 0.00	1.78	478	0.74
w54	0.63, 0.37, 0.00	1.24	594	1.00
w55	1.00, 0.00, 0.00	1.38	314	1.00
w57	1.00, 0.00, 0.00	1.42	718	1.00
w67	0.21, 0.79, 0.00	1.33	602	1.00
y80	0.86, 0.14, 0.00	1.34	1090	1.00
y81	0.88, 0.12, 0.00	1.10	859	0.42

p-values quantifying the goodness-of-fit of the power law distributions to the data. * denotes noisy erratic recordings.
