# Supplementary material for: Signatures of a globally optimal searching strategy in the three-dimensional foraging flights of bumblebees
Source: Sci Rep. 2016 Jul 27;6:30401. doi: 10.1038/srep30401 (PMC4961967; doi:10.1038/srep30401)
Supplement: Supplementary Information [file srep30401-s1.pdf]

Signatures of a globally optimal searching strategy in the three-dimensional foraging flights of bumblebees

Mathieu Lihoreau, Thomas C. Ings, Lars Chittka, Andy M. Reynolds

Supplementary Material

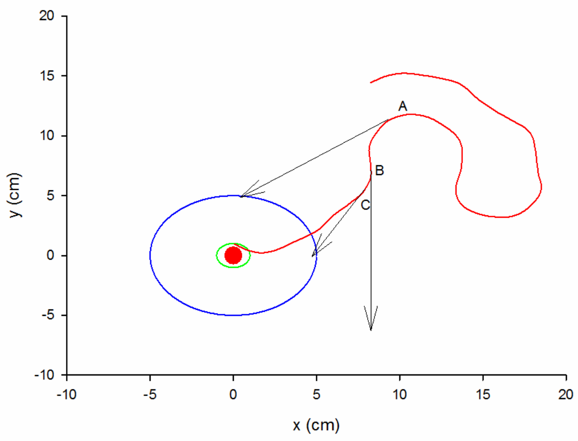

Figure S1 Schematic showing how detection ranges are calculated. An example synthetic flight pattern (red-line) that terminates within 1cm of a flower (green circle). The simulated bee is first heading towards the flower (i.e., would come within 5 cm of the flower (blue circle), if it maintained its current heading) at position ‘A’. But at this distance the bee was not locked onto the flower because it is later (at position B) heading away from the flower. After reaching position C it is continuously heading to within 5 cm of the flower, and so ‘locked’ onto the flower. The flower is therefore taken to have been detected at position C. Notice that after locking-on the flight pattern is not necessary straight.

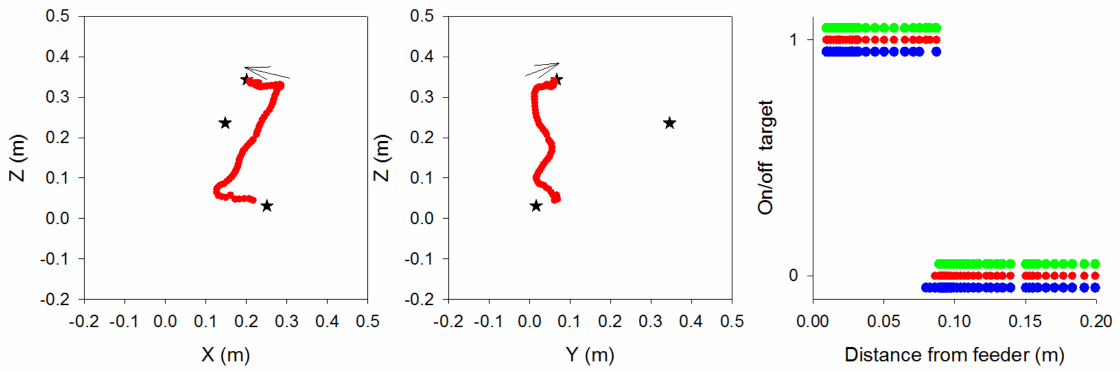

Figure S2 Partial recording of a flight pattern for bee 66 (bout 1) (●) illustrating how the detection range is calculated. The example flight pattern is projected onto the x-z and y-z planes. 100 recordings are shown made prior to the bee first coming within 1 cm a flower (*). Working back from the found feeder we find the furthest extent of continuous on-target flight, i.e., when all subsequent headings would bring the bee to within 5 cm of the feeder. This distance from the flower is taken to be the detection range. ‘Locking-on’ to a flower is here indicated by the arrows. In this example the detection range is about 8 cm. Comparable estimates for the detection range are obtained when the 5 cm threshold is replaced by 2.5 cm (●) and by 7.5 cm (●).

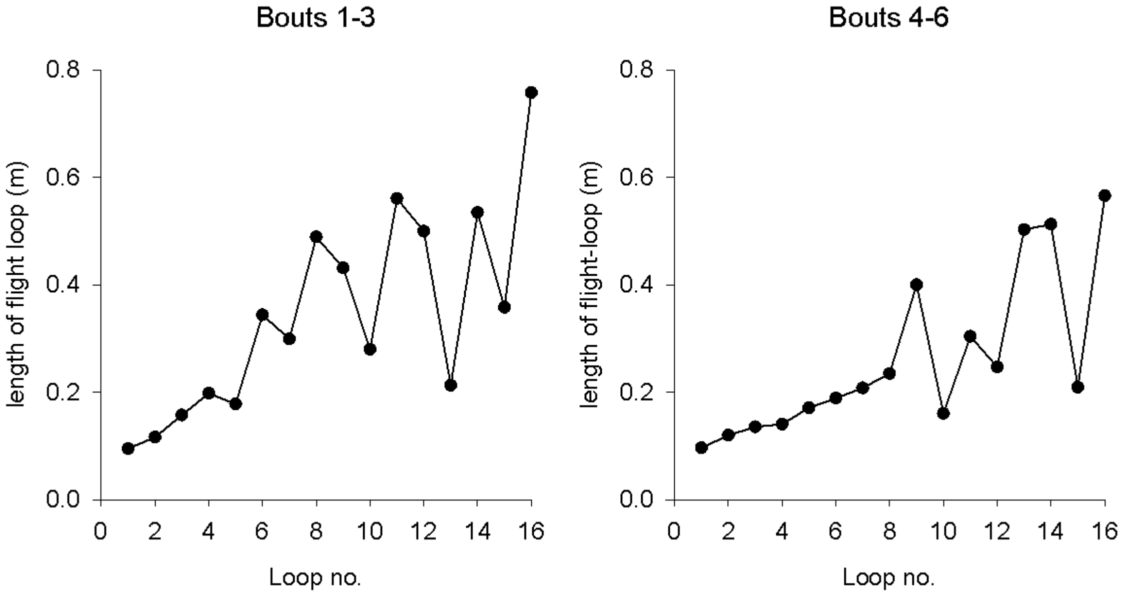

Figure S3 Quantification of loop length increase with time. Average loop lengths are shown as a function of the number of loops flown following a reset. Averages are shown for searching flights made in the presence of large (bouts 1-3) and small (bout 4-6) targets.

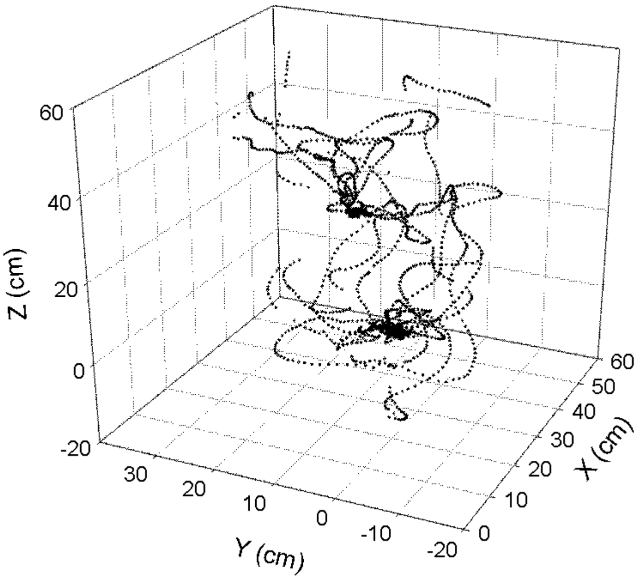

Figure S4 Complete three-dimensional recording of a flight pattern (bee b66 on bout 1).

Bee	Akaike weights for a power-law, a bi-exponential and a single exponential	Power-law exponent μ	No. of flight-steps	p-value for the power-law
b66	0.99, 0.01, 0.00	1.50	178	0.96
g84	0.92, 0.08, 0.00	1.67	248	1.00
y1	1.00, 1.00, 0.00	1.17	434	0.63
y2	0.00, 1.00, 0.00	1.21	336	-
w2	0.73, 0.27, 0.00	1.59	387	1.00
w11	0.99, 0.01, 0.00	1.50	178	0.96
w20	1.00, 0.00, 0.00	1.11	298	0.00
w49	0.00, 0.00, 1.00	1.00	753	0.00
w50	0.31, 0.69, 0.00	1.46	210	0.45
w54	0.28, 0.72, 0.00	1.30	319	1.00
w55	0.98, 0.02 ,0.00	1.48	287	1.00
w57	0.03, 0.97, 0.00	1.37	259	0.85
w67	0.98, 1.00, 0.00	1.50	305	0.97
y80	-	-	7281	*
y81	0.89, 0.11, 0.00	2.10	200	1.00

Table S1 Summary statistics for flight patterns of bees on their first foraging bouts in the presence of large flowers (bout 2). p-values quantifying the goodness-of-fit of the power law distributions to the data. * denotes noisy erratic recordings.

Bee	Akaike weights for a power-law, a bi-exponential and a single exponential	Power-law exponent μ	No. of flight-steps	p-value for the power-law
b66	1.00, 0.00, 0.00	1.67	503	1.00
g84	1.00, 0.00, 0.00	1.29	268	0.82
y1	0.65, 0.35, 0.00	1.25	708	0.35
y2	0.00, 1.00, 0.00	1.26	257	-
w2	1.00, 0.00, 0.00	1.56	513	1.00
w11	1.00, 0.00, 0.00	1.04	737	0.10
w20	0.65, 0.35, 0.00	1.27	300	0.76
w49	1.00, 0.00, 0.00	1.01	1418	0.02
w50	-	-	-	No data
w54	1.00, 0.00, 0.00	1.34	406	1.00
w55	0.97, 0.03 ,0.00	1.49	209	1.00
w57	0.00, 1.00, 0.00	1.76	653	-
w67	0.23, 0.77, 0.00	1.31	231	0.41
y80	1.00, 0.00, 0.00	1.43	223	0.02
y81	1.00, 0.00, 0.00	1.41	343	0.70-

Table S2 Summary statistics for flight patterns of bees on their first foraging bouts in the presence of large flowers (bout 3). p-values quantifying the goodness-of-fit of the power law distributions to the data. * denotes noisy erratic recordings.

Bee	Akaike weights for a power-law, a bi-exponential and a single exponential	Power-law exponent μ	No. of flight-steps	p-value for the power-law
b66	0.22, 0.78, 0.00	1.56	523	0.00
g84	-	-	7851	*
y1	0.00, 1,00, 0.00	1.25	658	-
y2	-	-	2125	*
w2	-	-	8603	*
w11	0.00, 0.00, 1.00	-	724	-
w20	1.00, 0.00, 0.00	1.18	569	0.71
w49	-	-	2825	*
w50	1.00, 0.00, 0.00	1.38	701	0.08
w54	0.36, 0.64, 0.00	1.27	727	1.00
w55	1.00, 0.00 ,0.00	1.41	484	1.00
w57	0.96, 0.04, 0.00	1.42	701	1.00
w67	0.00, 1.00, 0.00		648	-
y80	0.00, 1.00, 0.00		715	-
y81	-		1477	*

Table S3 Summary statistics for flight patterns of bees on their first foraging bouts in the presence of large flowers (bout 5). p-values quantifying the goodness-of-fit of the power law distributions to the data. * denotes noisy erratic recordings.

Bee	Akaike weights for a power-law, a bi-exponential and a single exponential	Power-law exponent μ	No. of flight-steps	p-value for the power-law
b66	0.02, 0.98, 0.00	1.30	575	0.11
g84	-	-	4855	*
y1	0.03, 0.97, 0.00	1.15	811	0.02
y2	0.00, 1.00, 0.00	1.03	512	-
w2	0.01, 0.99, 0.00	1.30	568	0.67
w11	0.00, 1.00, 0.00	-	1678	-
w20	0.00, 1.00, 0.00	-	1197	-
w49	-	-	4553	*
w50	0.04, 0.96, 0.00	1.62	586	0.00
w54	1.00, 0.00, 0.00	1.40	1170	1.00
w55	0.93, 0.00 ,0.00	1.37	372	0.87
w57	1.00, 0.00, 0.00	1.38	676	1.00
w67	-	-	1908	*
y80	1.00, 0.00, 0.00	1.37	447	0.50
y81	-	-	2884	-

Table S4 Summary statistics for flight patterns of bees on their first foraging bouts in the presence of large flowers (bout 6). p-values quantifying the goodness-of-fit of the power law distributions to the data. * denotes noisy erratic recordings
